# Hic-5 transduces mechanical force that drives a vicious cycle of bronchoconstriction

**DOI:** 10.21203/rs.3.rs-6498980/v1

**Published:** 2025-04-28

**Authors:** Chimwemwe Mwase, Wenjiang Deng, Hyo Jin Kim, Jennifer Mitchel, Thien-Khoi Phung, Michael J. O’Sullivan, Joel A. Mathews, Jeffrey Crosby, Christopher Turner, Adam Haber, Jin-Ah Park

**Affiliations:** Harvard T.H. Chan School of Public Health; Harvard T.H. Chan School of Public Health; Harvard T.H. Chan School of Public Health; Wesleyan University; Harvard T.H. Chan School of Public Health; Harvard T.H. Chan School of Public Health; Ionis Pharmaceuticals; Ionis Pharmaceuticals; SUNY Upstate Medical University; Department of Environmental Health, Harvard T.H. Chan School of Public Health, Boston, MA 02215, USA.; Harvard T.H. Chan School of Public Health

## Abstract

Mechanical forces are essential for the function of key organs, including the bladder, bowel, heart, and lung<1/sup>. These organs often encounter excessive or dysregulated mechanical forces, which are associated with pathological conditions. However, the key regulators of mechanotransduction remain poorly understood. As an example of how excessive mechanical force imposed on airway epithelia could lead to mechanotransduction<2/sup> that alters the transcriptome<3/sup> and secretome<4/sup> and induces cell death<5/sup>, all of which contribute to disease progression<6,7/sup>, we used human airway epithelial cells in air-liquid interface culture to mimic bronchoconstriction. We show that Hic-5, a focal adhesion adaptor protein, functions as a key regulator of mechanoresponses in the airway. Hic-5 expression is significantly induced in airway basal cells following mechanical compression or bronchoconstriction. Hic-5 knockdown using antisense oligonucleotides protects against stress fiber formation and abolishes approximately 70% of transcripts differentially regulated by mechanical compression. Moreover, Hic-5 deficiency attenuates secretion of ET-1, a potent bronchoconstrictor. Our data show that during an asthma exacerbation, Hic-5 reinforces a vicious cycle of bronchoconstriction through the secretion of ET-1. We establish Hic-5 as a critical link between mechanical stress and epithelial activation in human disease, implicating dysregulated mechanical forces as active drivers of disease progression with therapeutic relevance.

## Introduction

The confluent layer of endothelial and epithelial cells that lines the surfaces and lumens of internal organs is often exposed to excessive mechanical forces. These mechanical forces are linked to the pathophysiology of diseases in the heart, pulmonary aorta, esophagus, and lung. In the lung, distension during ventilator-induced lung injury and airway epithelial compression during asthma exacerbations have been recognized as contributors to disease progression. Specifically, bronchoconstriction during asthma exacerbations activates mechanotransduction in airway epithelial cells, leading to alterations in the transcriptome and secretome, as well as the induction of cell death^3–5^. These mechanically induced responses play a critical role in the progression of asthma^6,7^. Asthma exacerbations pose a significant clinical burden, contributing to hospitalizations, intensive care admissions, and approximately 461,000 deaths worldwide in 2019^8^. While most studies have focused on airway inflammation driven by immune cell activation, the contribution of bronchoconstriction is often underappreciated. However, as described above, emerging evidence indicates that the excessive mechanical forces generated during bronchoconstriction not only damage epithelial cells but also amplify airway inflammation and remodeling^5,7^. This mechanically initiated process may contribute to a vicious cycle that drives sustained and delayed asthmatic responses, the condition frequently experienced by patients with severe asthma^9^. Therefore, elucidating the precise mechanisms by which these mechanical forces are transduced into pathological responses in airway epithelial cells is essential for identifying new therapeutic targets and advancing the treatment of asthma. Moreover, uncovering how mechanical forces trigger inflammation and tissue remodeling likely has broader implications for disease in other organs where controlled mechanical forces are needed to maintain normal physiological functions.

To fill this gap, and as detailed in our experimental results, we investigated the role of Hic-5 (encoded by *TGFB1I1*), a focal adhesion adaptor protein^10,11^, in airway epithelial cells. Using RNA sequencing (RNA-seq) analysis of well-differentiated primary human bronchial epithelial (HBE) cells exposed to mechanical compression, mimicking epithelial deformation during bronchoconstriction, we identified *TGFB1I1* as a key mechanosensitive gene in airway epithelial cells. Hic-5 protein levels were also induced by mechanical compression, and the induction was dependent on the TGF-β receptor and ERK signaling. Single-cell RNA-seq (scRNA-seq) analysis of cells isolated from asthma patients revealed elevated *TGFB1I1* expression in basal airway epithelial cells and confirmed its link to bronchoconstriction. Hic-5 knockdown (KD) in HBE cells attenuated mechanoresponses, reducing differentially expressed genes (DEGs) by over 70%, including *EDN1*. This attenuation of *EDN1* expression led to decreased secretion of its encoded protein, endothelin-1 (ET-1), a potent bronchoconstrictor. Furthermore, Hic-5 KD protected against stress fiber formation, highlighting its role in the regulation of cellular force generation. These findings identify Hic-5 as a key regulator of airway epithelial mechanotransduction, linking mechanical stress to gene regulation and airway remodeling in asthma.

## Results

### TGFB1I1 expression is induced by mechanical compression and TGF-β1 in airway epithelial cells in vitro and during asthma exacerbations in humans

**Figure 1:**
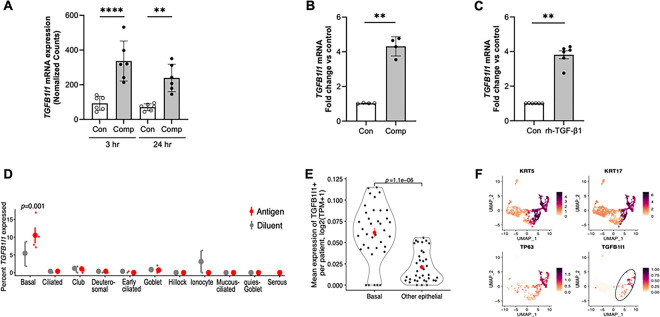
The expression of *TGFB1I1*, the Hic-5 encoding gene, is induced by mechanical compression, rhTGF-b1, and bronchoconstriction and detected in human airway basal cells. **(A)** Compression-induced *TGFB1I1* mRNA expression in HBE cells from scRNA-seq data. Expression plots show normalized *TGFB1I1* mRNA levels at 3 and 24 hours following mechanical compression. *TGFB1I1* mRNA expression was significantly increased by mechanical compression (mean ± SEM, n=6 HBE cell donors). ****p < 0.0001 and * p <0.05 vs vehicle control by one-way ANOVA. **(B)** *TGFB1I1* mRNA expression in HBE cells was measured at 24 hours post-compression. *TGFB1I1* mRNA expression was significantly increased by mechanical compression (mean ± SEM, n=4 HBE cell donors) **p < 0.001 vs vehicle control by paired t-test. (**C**) *TGFB1I1* mRNA expression in HBE cells was determined following exposure to rhTGF-b1 (10ng/ml) for 24 hours. Stimulation with rhTGF-b1 significantly increased *TGFB1I1* expression (mean ± SEM, n=6 HBE cell donors) ****p < 0.0001 vs vehicle control by paired t-test. **(D)** Re-analysis of published scRNA-seq data from human bronchial biopsies^16^. Visualizing the percentage of cells (y-axis) of each epithelial cell type (x-axis) in each patient (small dots) after challenge with an allergic antigen or diluent control (color legend). **(E)** Re-analysis of published scRNA-seq data from the Human Lung Cell Atlas^17^ comparing mean expression of *TGFB1I1* transcript (y-axis) in airway epithelial cells of each type (x-axis) in each individual donor (dots). (D-E) Large dots show the mean of all human donors, error bars: SEM. *P-*value: Wilcoxon rank-sum test. **(F)** Uniform Manifold Approximation and Projection (UMAP) embedding of published scRNA-seq profiles of human airway epithelial cells after 14 days of air-liquid interface (ALI) differentiation, downloaded from GEO accession (GSM2772992)^18^, colored by expression of key marker genes (color legends).

While the role of Hic-5 has been studied in the context of epithelial-to-mesenchymal transition (EMT) and cancer metastasis, its role in mechanically dysregulated lung conditions, particularly those associated with progressive pathological conditions, has not been previously explored, highlighting our novel findings^12–14^. In our RNA-seq analysis in HBE cells, we identified that mechanical compression significantly induced *TGFB1I1*, the gene encoding Hic-5 protein. In HBE cells from all six donors without underlying airway disease, *TGFB1I1* was significantly upregulated by mechanical compression at both 3and 24 hours post-compression (Fig. 1A). To validate our RNA-seq data, we performed a time-course experiment by measuring *TGFB1I1* mRNA expression by qPCR at 3, 8, and 24 hours post-compression. We observed a transient increase in *TGFB1I1* mRNA expression, which was maximally induced at 3 hours post-compression, with its significant induction of 4.2-fold (p < 0.001, Fig. 1B) compared to the time-matched control. As *TGFB1I1* was originally identified as a TGF-β1-inducible gene, and its induction by TGF-β1 has been observed in multiple cell types^15^, we tested whether it is similarly induced by TGF-β1 in HBE cells, given that TGF-β1 levels are elevated in patients with asthma. After incubation of HBE cells with recombinant human TGF-β1 (rh-TGF-β1, 10 ng/ml) for 24 hours, we detected a significant 3.5-fold induction of *TGFB1I1* mRNA expression (p < 0.0001, Fig. 1C).

To further establish a mechanistic link between *TGFB1I1* induction in our *in vitro* system and its clinical relevance during bronchoconstriction in asthma exacerbations, we employed a novel approach by annotating *TGFB1I1 expression using* scRNA-seq data from published human asthma exacerbation studies^16^. This analysis revealed a significant induction of *TGFB1I1* expression, predominantly in basal airway epithelial cells, following allergen challenge (Fig. 1D). Importantly, this induction was observed exclusively in individuals with asthma experiencing bronchoconstriction, whereas it was absent in allergic individuals without asthma. We then validated this cell-type-specific expression of *TGFB1I1* in basal cells by analyzing two additional publicly available scRNA-seq datasets^17,18^. In the first scRNA-seq dataset^17^, we assessed the spatial distribution of *TGFB1I1* expression across the respiratory tract, including the nasal passages, airways, and lung parenchyma (**Extended Data Fig. 1**). Our analysis demonstrated the expression of *TGFB1I1* in both the airway and lung parenchyma, but not in the nasal passages. Within the airway, *TGFB1I1* expression was significantly higher in basal cells compared to non-basal cell populations (Fig. 1E). In the second dataset^18^, which is well-defined scRNA-seq data from the Human Cell Atlas, *TGFB1I1* expression was enriched in cells annotated by basal cell-specific markers, such as *TP63*, *KRT5*, and *KRT17* (Fig. 1F). Both of the analyses confirmed a predominant expression of *TGFB1I1* in basal cells in human airways.

### Hic-5 protein induction is mediated by the TGF-β receptor-ERK axis in response to mechanical compression and TGF-β1

**Figure 2:**
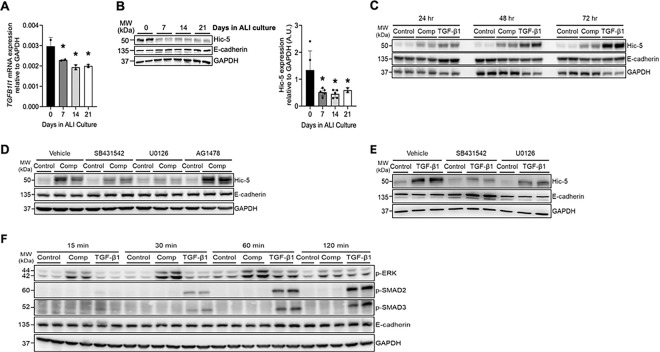
The induction of Hic-5 depends on the activation of TGF-β receptor and ERK. **(A)** *TGFB1I1* mRNA expression progressively decreased as basal cells differentiated over the course of 21 days in ALI culture. *p < 0.05, by one-way ANOVA with Holm-Šídák post-hoc correction. **(B)** Representative western blots present that Hic-5 protein was prominently detectable in confluent basal cell layers on ALI day 0, and its expression decreased as the basal cells differentiated. Densitometry analysis was performed across five biological replicates (n= 5 HBE cell donors), and the fold change relative to GAPDH was calculated [arbitrary units (A.U.)]. *p < 0.05, by one-way ANOVA with Holm-Šídák post-hoc correction. (**C**) In well-differentiated HBE cells, Hic-5 protein increased in response to both mechanical compression and rhTGF-β1 treatment. Compression-induced Hic-5 protein expression remained constant for up to 72 hours post-compression, whereas rhTGF-β1 stimulation progressively increased Hic-5 protein levels over the same time period. (**D**) Representative western blots demonstrate that the compression-induced Hic-5 expression was attenuated by SB431542 (TGF-β receptor inhibitor) and U0126 (ERK inhibitor) but remained unaffected by AG1478 (EGFR inhibitor). E-cadherin and GAPDH were detected as loading controls (n=2 HBE cell donors). (**E**) Representative western blots demonstrate that the TGF-β1-induced Hic-5 expression was attenuated by SB431542 and U0126. E-cadherin and GAPDH were detected as loading controls (n=2 HBE cell donors). (**F**) Representative western blots present the time course of phosphorylation of SMAD2, SMAD3, and ERK (p-SMAD2, p-SMAD3, and p-ERK) in HBE cells following stimulation with mechanical compression or TGF-β1. Compression prominently increased p-ERK levels as early as 15 minutes and sustained up to 60 minutes post-stimulation but did not induce either p-SMAD2 or p-SMAD3. TGF-β1 substantially increased p-SMAD2 and p-SMAD3 within 30 minutes and sustained up to 120 minutes, while modestly increased p-ERK levels detected at 60 minutes (n=2 HBE cell donors).

Consistent with *TGFB1I1* being annotated in basal cells, our qPCR analysis also detected a substantial level of *TGFB1I1 mRNA expression* at the time the air-liquid interface (ALI) culture was established (on ALI day 0), which subsequently decreased over the course of differentiation in ALI culture (Fig. 2A). Unlike this reduction during differentiation, expression of the basal cell marker gene *TP63* remained unchanged, whereas expression of differentiation markers, *MUC5AC*, *FOXJ1*, and *DNAI2*, increased (**Extended Data Fig. 2**). Hic-5, the protein encoded by *TGFB1I1*, was detectable by western blot analysis in confluent basal cell layers but significantly decreased over the course of ALI culture (Fig. 2B). Similar to the induction of *TGFB1I1* expression (Fig. 1A, B), Hic-5 protein expression was induced by both mechanical compression and TGF-β1 (Fig. 2C). The induction of Hic-5 by mechanical compression was detected at the first 24 hours post-compression and remained constant with no further induction up to 72 hours. In contrast, the induction of Hic-5 by TGF-β1, which was detected at 24 hours continued to increase in a time-dependent manner, showing further elevation at 48 and 72 hours (Fig. 2C). These differences in the kinetics of Hic-5 protein induction may be attributed to the source of TGF-β1, with autocrine production by mechanically compressed epithelial cells versus exogenous TGF-β1 treatment, which mimics its presence in the extracellular environment under chronic disease conditions.

To elucidate the signaling pathways regulating the induction of the Hic-5 protein, we tested inhibitors targeting key signaling molecules activated by mechanical compression, including the TGF-β receptor, ERK, and EGFR, which we previously identified^2,19–21^. To block each pathway in mechanically compressed cells, we pretreated the cells with pharmacological inhibitors: SB431542 for the TGF-β receptor, U0126 for ERK, and AG1478 for EGFR. Pretreatment with SB431542 and U0126 attenuated compression-induced Hic-5 protein expression, indicating that compression-induced Hic-5 expression was dependent on the ERK and TGF-β receptor pathways. In contrast, pretreatment with AG1478 had no effect, indicating that compression-induced Hic-5 expression was independent of the EGFR pathway (Fig. 2D). Similarly, pretreatment with SB431542 and U0126 attenuated TGF-β1-induced Hic-5 expression (Fig. 2E), confirming that the TGF-β receptor-ERK axis serves as a common mechanism for Hic-5 induction in response to either mechanical compression or TGF-β1. Given that the TGF-β receptor-ERK axis mediates Hic-5 activation under both conditions, we further examined the downstream TGF-β receptor pathways activated by compression and TGF-β1. We detected p-SMAD2 and p-SMAD3, indicative of the canonical TGF-β receptor pathway, and p-ERK, indicative of the non-canonical TGF-β receptor pathway (Fig. 2F). As expected, TGF-β1 treatment induced both p-SMAD2 and p-SMAD3 within 30 minutes, followed by a moderate increase in p-ERK at later time points. In contrast, compression markedly induced p-ERK with no noticeable induction of either p-SMAD2 or p-SMAD3.

### Hic-5 regulates mechanoresponsive genes in HBE cells

**Figure 3:**
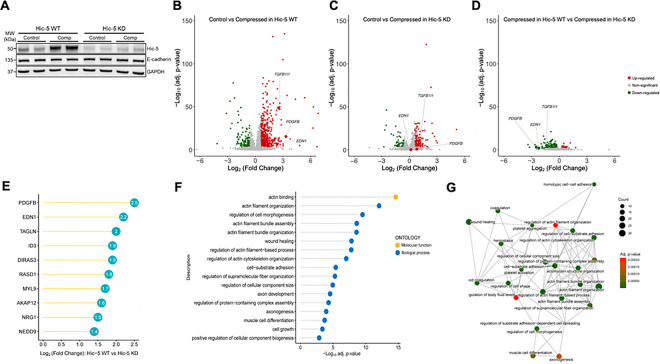
Mechanical compression regulates multiples cellular responses in a Hic-5-dependent manner. **(A)** Representative western blots demonstrate the effective knockdown of Hic-5 in bronchial epithelial cells at baseline and in response to mechanical compression. Compression induced Hic-5 expression in Hic-5 wildtype (WT) cells, whereas this Hic-5 induction was abolished in Hic-5 knockdown (KD) cells. n > 6 HBE cell donors **(B–C)** Volcano plots present differentially expressed genes (DEGs) between control and compressed conditions. In Hic-5 WT cells, mechanical compression induced a significant number of DEGs (B). In Hic-5 KD cells, the number of DEGs were significantly fewer compared to WT cells (C). n=3 HBE cell donors. **(D)** The volcano plot presents DEGs between Hic-5 WT and Hic-5 KD cells under compression. In B-D, up-regulated genes are marked by red, down-regulated genes by green, and not significant genes by gray. **(E)** The top 10 DEGs were ranked by Log_2_ (Fold Change) between Hic-5 WT vs Hic-5-KD under compression. **(F)** Gene Ontology (GO) analysis identified biological processes and molecular functions of up-regulated genes in Hic-5 WT cells compared with Hic-5 KD under compression as demonstrated in (**D**). **(G)** The enrichment network visualized functional modules of up-regulated genes in Hic-5 WT cells compared with Hic-5 KD under compression as demonstrated in (**D**).

Our data, as demonstrated in Fig. 2, indicated that Hic-5 induction depended on key signaling pathways regulating asthma-associated mediators^2,19–21^. Given these mediators are induced by mechanical compression in HBE cells, we hypothesized that Hic-5 plays a regulatory role in airway epithelial mechano-responses. To test this, we used an antisense oligonucleotide (ASO)-mediated knockdown approach in well-differentiated HBE cells. Among the five ASO sets we tested in primary HBE cells cultured from multiple donors, we selected the ASO set demonstrating the highest knockdown efficiency of Hic-5, both at baseline and in response to mechanical compression (**Extended Data Fig. 3**). In HBE cells treated with a control (scrambled) ASO (denoted as Hic-5 WT), mechanical compression substantially induced Hic-5 protein levels (Fig. 3A). In contrast, in HBE cells treated with an ASO targeting Hic-5 (denoted as Hic-5 KD), where Hic-5 protein levels were markedly lower than in Hic-5 WT cells, the compression-induced increase in Hic-5 protein was minimal, resulting in levels even lower than those detected under control conditions in Hic-5 WT cells (Fig. 3A). Using this ASO-mediated KD approach combined with bulk RNA-sequencing analysis, we next investigated how Hic-5 deficiency modulates transcriptional profiles in HBE cells at baseline and in response to mechanical compression. In both WT (Fig. 3B) and Hic-5 KD (Fig. 3C) HBE cells, we identified differentially expressed genes (DEGs) between control and compressed conditions at 3hr post-compression. In WT HBE cells, 445 genes were up-regulated, and 159 genes were down-regulated (absolute log_2_-fold change > 0.6 and FDR < 0.001, Fig. 3B). In contrast, Hic-5 KD cells displayed a markedly attenuated response to compression, with only 136 upregulated and 49 downregulated genes (Fig. 3C). *TGFB1I1* expression was significantly increased by 5.7-fold (FDR < 0.001) in Hic-5 WT. While its expression appeared to be significantly induced by 1.2-fold in Hic-5 KD cells, this was likely due to a substantial reduction in *TGFB1I1* baseline levels, as shown in Fig. 3A. To visually compare how mechanoresponsive genes were differentially regulated by mechanical compression in Hic-5 WT and Hic-5 KD, we also illustrated the DEGs by volcano plot (Fig. 3D) and heatmaps (**Extended Data Fig. 4**). The results indicated that 193 genes were downregulated in Hic-5 KD compared with the Hic-5 WT. This confirmed that *TGFB1I1* was one of the downregulated genes as annotated in Fig. 3D. To identify candidate genes regulated by Hic-5, we ranked the downregulated genes by log_2_ fold-change values (Fig. 3E). The top-ranked genes included *PDGFB*, which had a log_2_ fold-change of −2.5 in Hic-5 KD cells, followed by *EDN1* with a log_2_ fold-change of −2.2. In Fig. 3B and Fig. 3C, we annotated *PDGFB* and *EDN1* to highlight their responses to mechanical compression in Hic-5 WT and Hic-5 KD HBE cells. In WT, *PDGFB* and *EDN1* showed significant increases of 9.9- and 5.3-fold, respectively (Fig. 3B). However, in Hic-5 KD cells, the expression of both genes showed no significant differences between control and compressed cells (Fig. 3C and 3D). To explore the biological pathways affected by Hic-5 KD, we performed gene ontology (GO) analysis using the down-regulated genes (Fig. 3F). The pathways modulated by Hic-5 KD include actin binding, actin cytoskeleton organization, and regulation of cell morphogenesis, all of which are related to cellular differentiation, morphogenesis, and migration. To examine the overlap and potential interactions between these activated pathways, we generated a gene enrichment network plot (Fig. 3G), which enabled more detailed analysis of the pathway modules affected by Hic-5 KD. The first module involved actin filament and structure programs, including the genes *NEDD9*, *LCP1*, and *ACTN1*, suggesting that Hic-5 KD may disturb the normal actin filament functions ^22^. The second module involved cell shape regulation, cell-substrate adhesion, and wound healing-related programs, including the genes *PDGFB, EDN1*, and *FERMT2*, implicating that Hic-5 may play a role in maintaining the structural integrity of the airway barrier^23^. Lastly, the third major module involved platelet activation, hemostasis, and coagulation programs, including the genes *PDGFB*, *EDN1*, and *F3*, indicating that Hic-5 is needed for hemostatic activity in the lung^24^.

#### Hic-5 deficiency protects against stress fiber formation and cell shape change in response to mechanical compression

**Figure 4:**
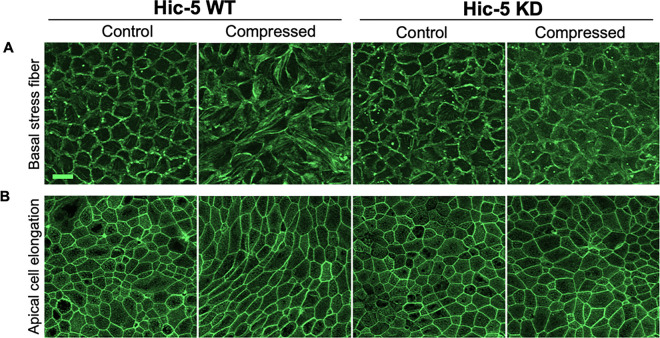
Hic-5 drives basal cell stress fiber formation and apical cell elongation in response to mechanical compression. Representative immunofluorescence (IF) images of F-actin in Hic-5 WT and Hic-5 KD HBE cells at 24 hours post-compression. Mechanical compression induced basal cell stress fiber formation **(A)** and apical cell elongation **(B)** in Hic-5 WT HBE cells but not in Hic-5 KD HBE cells. Scale bar = 20 mm, n=5 HBE cell donors.

We have previously demonstrated that mechanical compression induces basal cell stress fiber formation that is associated with intercellular force generation during epithelial cell unjamming transition^25^. This stress fiber formation could be regulated by Hic-5 as indicated in our gene ontology analysis (Fig. 3F). To validate the role of Hic-5 in stress fiber formation, we stained F-actin in both Hic-5 WT and KD cells with and without compression. Consistent with our previous findings, in Hic-5 WT, compression induced basal stress fiber formation (Fig. 4A). However, in Hic-5 KD HBE cells, this compression-induced stress fiber formation was abolished. Furthermore, Hic-5 deficiency protected against compress-induced epithelial elongation as observed in apical sides of pseudostratified epithelium (Fig. 4B).

#### Hic-5 deficiency inhibits endothelin-1 secretion

**Figure 5:**
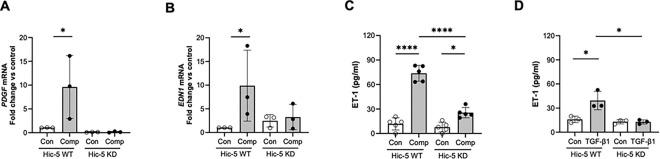
Endothelin-1 expression and secretion depend on Hic-5. **(A-B)** The validation of the top 2 ranked DE genes between Hic-5 WT and Hic-KD were quantified by qPCR. Compression-induced expression of *PDGF* (**A**) and *EDN1* (**B**) was observed in Hic-5 WT HBE cells but was abolished in Hic-5 KD cells (mean ±SD, n=3 HBE cell donors). *p < 0.05, one-way ANOVA with Bonferroni post-hoc correction. **(C-D)** Secretion of endothelin-1 (ET-1) by HBE cells into the basolateral conditioned media was determined by ELISA at 24 hours post-compression (**C**) and post-TGF-β1 treatment **(D)**. Hic-5 deficiency (Hic-5 KD) attenuated ET-1 secretion that was induced by both compression and TGF-β1 (mean ± SD, n=5 HBE cell donors in **C** and n=3 HBE cell donors in **D**). *p < 0.05 and ****p < 0.0001, one-way ANOVA with Bonferroni post-hoc correction.

We then further determined the role of Hic-5 in the regulation of *PDGFB* and *EDN1,* two top-ranked genes, as remonstrated in our RNA-seq analysis (Fig. 3E). We quantified *PDGFB* mRNA expression by qPCR in both WT and KD HBE cells. In Hic-5 WT HBE cells, mechanical compression induced *PDGFB* mRNA expression by 9.6-fold (*p < 0.05) compared to control (Fig. 5A). However, in Hic-5 KD HBE cells, compression-induced *PDGFB* expression was completely abolished. PDGF is known to be secreted from various cell types, including epithelial cells and detected at high concentrations in asthma^26,27^. Therefore, we also assessed the effect of Hic-5 deficiency on the secreted PDGF protein levels. However, secreted PDGF protein was undetectable by ELISA (R&D ELISA kit) under all conditions. We next validated *EDN1*, the second top-ranked gene highlighted in Fig. 3E, which encodes ET-1 protein. This validation was particularly intriguing, as we previously reported that mechanical compression induces ET-1 protein secretion, which in turn stimulates airway smooth muscle (ASM) contraction^28^. Consistent with our prior findings, in Hic-5 WT HBE cells, mechanical compression significantly induced *EDN1* mRNA expression by 9.9-fold (*p < 0.05, Fig. 5B) and increased ET-1 secretion by 84% (****p < 0.0001, Fig. 5C) compared to control. However, in Hic-5 KD HBE cells, the compression-induced *EDN1* mRNA expression was significantly reduced by 67% (3.26-fold) and ET-1 secretion was similarly decreased by 65% (****p < 0.0001) compared to the compressed condition in Hic-5 WT cells (Fig. 5B and 5C). Moreover, ET-1 secretion induced by TGF-β1 in Hic-5 WT was also significantly abolished in Hic-5 KD (*p < 0.05, Fig. 5D).

## Discussion

In this study, we identified Hic-5, encoded by *TGFB1I1*, as a key regulator of mechanotransduction in human airway epithelial cells. Using bulk RNA-seq in well-differentiated HBE cells and reanalyzing published scRNA-seq data from human bronchial biopsies collected after allergen challenge^16^, we established Hic-5 as a link between our in vitro findings and bronchoconstriction-induced epithelial responses in patients with asthma. Using HBE cells exposed to mechanical compression mimicking airway epithelial cell deformation during bronchoconstriction, we demonstrated that Hic-5 was induced through pathways dependent on the TGF-β receptor and ERK. Functional analysis using Hic-5 KD combined with RNA-seq revealed a significant reduction in DEGs between control and compressed HBE cells, indicating that Hic-5 plays a key role in transcriptional regulation under mechanical stress. Our data indicate that Hic-5 mediates key compression-induced responses, including actin cytoskeleton organization and ET-1 secretion, highlighting its role as a key link between mechanical stress on the airways and ASM contraction, a critical pathological component of asthma.

Our findings have important therapeutic implications for asthma treatment. Hic-5, a member of the paxillin superfamily, functions as a focal adhesion scaffold that transduces mechanical signals from extracellular stimuli to intracellular signaling cascades^11,29,30^. Hic-5 has been recognized as a regulator of cellular mechanoresponses in mostly mesenchymal or cancerous cells^12–14,29,31–34^. While its superfamily member protein paxillin has been studied for its role for epithelial injury responses through focal adhesion remodeling^35^, the role of Hic-5 in epithelial tissues remains poorly defined. This limited understanding may be due to its low expression in mature epithelial cells, with levels increasing during EMT^13,36^. Consequently, Hic-5 has been studied more extensively in the context of cancer, where the EMT is a prominent feature^36–38^. Our novel findings move this field forward by identifying that mechanically induced Hic-5 initiates a cascade of events leading to airway constriction, a key component of the asthmatic response.

Our RNA-seq and qPCR analyses confirmed that mechanical compression significantly induced *TGFB1I1* expression in well-differentiated primary HBE cells (Figs. 1 and 2). Since TGF-β1 signaling regulates epithelial proliferation, differentiation, extracellular matrix production, and inflammation, all of which contribute to asthma pathogenesis^39–43^, Hic-5 may serve as a molecular bridge between mechanical and biochemical signaling in asthma. Elevated TGF-β1 levels in BALF, airway epithelium, and eosinophils correlate with disease severity^41,42,44^, and TGF-β receptor activation contributes to airway remodeling and airway hyperresponsiveness (AHR) in both mice and humans^7,45,46^. Therefore, we hypothesized that Hic-5 acts as a key link between TGF-β receptor activation and cellular responses to both mechanical compression and TGF-β1 stimulation. scRNA-seq data revealed that *TGFB1I1* is enriched in basal cells, a progenitor population critical for airway repair and remodeling. Furthermore, *TGFB1I1* expression increased in basal cells of asthma patients following allergen exposure^16^, linking Hic-5 to airway narrowing and remodeling.

In ALI cultures, Hic-5 protein levels decreased as basal stem cells differentiated, suggesting its potential roles in basal cell homeostasis, epithelial differentiation, and regeneration. Although well-differentiated HBE cells exhibited low baseline Hic-5 expression, it became inducible by mechanical compression and TGF-β1 in a manner dependent on TGF-β receptor and ERK signaling (Fig. 2). Interestingly, although our previous work demonstrated that the EGFR–ERK axis is required for YKL-40 and EGF ligand expression in mechanically compressed cells, Hic-5 induction is independent of EGFR activation^2,19^. Given this apparent disconnection of EGFR and ERK in Hic-5 regulation, we further explored whether ERK activation occurs through a TGF-βR-dependent pathway. TGF-β receptor signaling operates through both canonical (SMAD2/3) and non-canonical (ERK, JNK, MAPK, and ROCK) pathways^43^. In HBE cells, TGF-β1 stimulation predominantly activated the canonical SMAD2/3 pathway, while mechanical compression preferentially triggered non-canonical TGF-β-ERK signaling, despite both conditions converging on Hic-5 induction. Notably, TGF-β1–induced Hic-5 has been shown to be essential for sustained TGF-β1 production through a feed-forward mechanism, in which persistently high TGF-β1 levels maintain the pathogenic myofibroblast phenotype observed in hypertrophic scar tissue^47^.

Our GO and gene enrichment network analyses revealed that Hic-5 regulates key cellular responses to mechanical stress, including actin filament organization, cell shape, wound healing, and coagulation activity (Fig. 3). In a previous report using trabecular meshwork cells, TGF-β2 and ET-1 are shown to induce Hic-5 expression, which in turn regulates actin cytoskeleton organization^12^. In our study, we observed that Hic-5 deficiency disrupted stress fiber formation, supporting its role in the dynamic regulation of cytoskeleton organization and cell shape changes. Stress fiber formation governs cellular responses to mechanical stress by increasing stiffness and promoting cell shape elongation, thereby increasing epithelial susceptibility to mechanical injury^35,48,49^. In A549 cells, transmural pressure leads to the activation of NFκB in a manner dependent on actin stress fiber formation, indicating a potential role of the actin cytoskeleton in mechanotransduction-mediated inflammatory signaling^50^. This may underlie the mechanism of epithelial cell death and subsequent inflammation observed in models of bronchoconstriction^5^. The association between Hic-5 and cell shape elongation is also reported in cancer cells^33^, suggesting a broader role for Hic-5 in regulating cellular responses. The most impactful finding of this study is that Hic-5 reinforces a vicious cycle in which bronchoconstriction-induced mechanical stress triggers ET-1 secretion, thereby promoting late asthmatic responses with sustained airway narrowing frequently experienced by patients with severe asthma.

Our study identifies Hic-5 as a key regulator of mechanotransduction in airway epithelial cells, linking bronchoconstriction-induced mechanical stress to asthma pathogenesis. Through its roles in cytoskeleton organization and transcriptional regulation, including ET-1, Hic-5 links acute asthma exacerbations to sustained asthmatic responses and airway remodeling, the hallmark of difficult-to-treat asthma. Together, our findings provide new mechanistic insights into how mechanical stress shapes epithelial responses and highlight Hic-5 as a potential therapeutic target for mitigating disease progression in asthma. Moreover, our data extend our understanding of how dysregulated chronic mechanical stress promote the progression of disease, offering novel perspectives applicable to other mechanobiology-driven conditions.

## MATERIALS AND METHODS

### Reanalysis of published single cell RNA seq data

Previously published scRNA-seq data from human asthma challenge model^1^, human lung cell atlas^2^, and human in vitro cultured airway epithelial cells^3^ were downloaded from the respective relevant online repository and visualized using standard computational approaches.

### Air-liquid interface (ALI) culture of primary human bronchial epithelial (HBE) cells

As previously described ^4–8^, primary HBE cells were seeded on transwells and maintained in ALI culture until the cells were well-differentiated. Primary HBE cells were isolated at passage 0 at the Marsico Lung Institute/Cystic Fibrosis Research Center, the University of North Carolina (UNC) at Chapel Hill. Donor lungs were obtained under a protocol approved by the Institutional Review Board of the UNC, Chapel Hill (approval no. 03–1396). Informed consent was obtained from legally authorized representatives of all organ donors. We used primary HBE cells from thirteen distinct donors with no history of smoking or lung disease.

### In vitro exposure of HBE cells to mechanical compression, TGF-β1, or inhibitors

To mimic bronchoconstriction, well-differentiated HBE cells were exposed to apical-to-basal pressure with a magnitude of 30 cm H_2_O for 3 hours, as previously described ^4–8^. Time-matched control cells received 0 cm H_2_O pressure. Cells and conditioned media were collected between 3 and 72 hours. In experiments using recombinant human (rh) TGF-β1 (Cell Signaling Technologies, Danvers, MA), we spiked 10 ng/ml into the basolateral media of HBE cells in ALI cultures. Cells were collected between 24 and 72 hours for further analysis. To determine the signaling pathways that regulate mechanical compression-induced Hic-5 protein expression, we used pharmacological inhibitors of the TGF-β receptor (SB431542; 10 μM, Tocris, Bristol, UK), MEK (U0126; 10 μM, Tocris), and EGFR (AG1478; 10 μM, Tocris). Each inhibitor was added to the basolateral media of HBE cells 1 hour prior to exposure to mechanical compression or TGF-β1. As a control, 0.1% DMSO was used.

### Hic-5 knockdown using antisense oligonucleotides (ASO)

To knock down Hic-5 expression in HBE cells, we used antisense oligonucleotides designed and provided by Ionis Pharmaceuticals. We initially tested five ASOs targeting *TGFB1I1*(denoted as Hic-5 ASO). Then, we selected the most effective ASO and used for subsequent experiments. HBE cells maintained in ALI culture were treated with 10μM of either a non-targeting control (scrambled) ASO or Hic-5 ASO between ALI days 9 and 20 prior to the application of mechanical compression. We confirmed the knockdown efficiency of the Hic-5 ASO in well-differentiated HBE cells by RT-qPCR and western blot analysis.

### RNA-Sequencing analysis

As we previously described ^4–8^, we isolated total RNA from HBE cells (n = 3 donors without a history of lung disease) using the RNeasy Mini Kit (Qiagen, Hilden, Germany). Bulk RNA sequencing was performed on a NovaSeq instrument (Illumina, San Diego, CA, USA) by the Bauer Core Facility at Harvard University.

We evaluated the quality of RNA-seq data using MultiQC (v1.9) and FastQC (v0.12.0), and filtered out sequencing reads with low-quality metrics. Transcript expression at the isoform level was quantified using Salmon (v1.10.1), and gene-level expression values were then aggregated from the isoform-level results. Differentially expressed genes (DEGs) between the control and compressed groups were identified using DESeq2 (v1.42.0), enabling the detection of both up-regulated and down-regulated genes between Hic5-WT and Hic5-KD samples. DEGs were defined as adjusted p-value < 0.05 and an absolute Log2(Fold Change) > 0.25. The DEG results were visualized using volcano plots and heatmaps. Additionally, DESeq2 was employed to assess interaction effects between genotypes (i.e. Hic-5 WT vs. Hic-5 KD) and experimental conditions (i.e. control vs. compression). Gene ontology (GO) and pathway enrichment analyses were performed on significant DEGs from each comparison using clusterProfiler (v4.8.2). To facilitate efficient identification of functional modules, gene enrichment networks were constructed with the enrichplot package (v1.26.5). All data analyses and visualizations were conducted using R (v4.3.2) in RStudio (v2023.09.1).

### RT-qPCR

We performed real-time RT-qPCR using the primers in the table below as previously described ^4,8^. After normalization to *GAPDH*, the fold changes were calculated by the comparative 2^−ΔΔCt^ method ^9^. To compare mRNA expression relative to *GAPDH* over the course of ALI culture, we calculated 2^−ΔCt^.

### Western blot analysis

We detected proteins in cell lysates by western blot analysis as previously described ^8^. We used the following primary antibodies according to the manufacturer’s instructions: Hic-5 (#6114; BD Biosciences, Woburn, MA), (E-cadherin (#3195), GAPDH (#5174S), p-ERK (#4370S), p-SMAD2 (#3108), p-SMAD3 (#9520); Cell Signaling Technology, Danvers, MA). GAPDH was detected as the loading control. Protein intensity was analyzed using Image J software (v1.46; National Institutes of Health, Bethesda, MD, USA).

### Immunofluorescence staining

Using our previously described immunofluorescence staining protocol ^12^, we stained cells for F-actin using phalloidin conjugated to AlexaFluor 488 (A12379; ThermoFisher Scientific, Waltham, MA), at 24hrs after compression. Cells were counterstained with Hoescht (33342; ThermoFisher Scientific) for nuclei.

### ELISA

We measured the amount of ET-1 protein released into basolateral conditioned media using a human specific ELISA kit (DET100; R&D Systems, Minneapolis, MN), according to the vendor’s instructions.

### Statistical analysis

All the data were analyzed using GraphPad Prism (San Diego, CA). Statistical significance was determined using a two-tailed Student’s t-test in experiments comparing two groups, or ANOVA followed by Bonferroni or Holm-Šídák post-hoc corrections in experiments comparing three or more groups. A *p* < 0.05 was considered statistically significant.

## Figures and Tables

**Table 1.** Primer sequences used in RT-qPCR

Gene	Primers	Ref
*GAPDH*	FW 5’-TGGGCTACACTGAGCACCAG-3’ RV 5’-GGGTGTCGCTGTTGAAGTCA-3’	4
*EDN1*	FW 5’-AGAGTGTGTCTACTT CT GCCA-3’ RV 5’-CTTCCAAGTCCATACGGAACAA-3’	5
*TGFB1I1*	FW 5’- GACTTCCTGCAGCTGTTCG-3’ RV 5’- AAGTGGTTCTCGCACAACG-3’	10
*PDGFB*	FW 5’- ACTCGATCCGCTCCTTTGATGA-3’ RV 5’- GCTCGCCTCCAGAGTGGG-3’	11

## Data Availability

Data that comprise the graphs within this manuscript and other findings of this study are available from the corresponding author upon request.
